# A metastable subproteome underlies inclusion formation in muscle proteinopathies

**DOI:** 10.1186/s40478-019-0853-9

**Published:** 2019-12-03

**Authors:** Prajwal Ciryam, Matthew Antalek, Fernando Cid, Gian Gaetano Tartaglia, Christopher M. Dobson, Anne-Katrin Guettsches, Britta Eggers, Matthias Vorgerd, Katrin Marcus, Rudolf A. Kley, Richard I. Morimoto, Michele Vendruscolo, Conrad C. Weihl

**Affiliations:** 1 Department of Neurology, Vagelos College of Physicians & Surgeons, Columbia University, New York, NY USA; 2 Rice Institute for Biomedical Research, Department of Molecular Biosciences, Northwestern University, Evanston, IL USA; 3 Centre for Genomic Regulation, The Barcelona Institute of Science and Technology, Barcelona, Spain; 4 Centre for Misfolding Diseases, Department of Chemistry, University of Cambridge, Cambridge, UK; 5 Department of Neurology, Heimer Institute of Muscle Research, University Hospital Bergmannsheil, Ruhr-University Bochum, Bochum, Germany; 6 Medizinisches Proteom-Center, Ruhr-University Bochum, Bochum, Germany; 7 Department of Neurology, St. Marien Hospital Borken, University of Witten/Herdecke, Borken, Germany; 8 Department of Neurology and Hope Center for Neurological Disease, Washington University School of Medicine, Saint Louis, MO USA

## Abstract

Protein aggregation is a pathological feature of neurodegenerative disorders. We previously demonstrated that protein inclusions in the brain are composed of supersaturated proteins, which are abundant and aggregation-prone, and form a metastable subproteome. It is not yet clear, however, whether this phenomenon is also associated with non-neuronal protein conformational disorders. To respond to this question, we analyzed proteomic datasets from biopsies of patients with genetic and acquired protein aggregate myopathy (PAM) by quantifying the changes in composition, concentration and aggregation propensity of proteins in the fibers containing inclusions and those surrounding them. We found that a metastable subproteome is present in skeletal muscle from healthy patients. The expression of this subproteome escalate as proteomic samples are taken more proximal to the pathologic inclusion, eventually exceeding its solubility limits and aggregating. While most supersaturated proteins decrease or maintain steady abundance across healthy fibers and inclusion-containing fibers, proteins within the metastable subproteome rise in abundance, suggesting that they escape regulation. Taken together, our results show in the context of a human conformational disorder that the supersaturation of a metastable subproteome underlies widespread aggregation and correlates with the histopathological state of the tissue.

## Introduction

The presence of protein aggregates is a hallmark of many age-related degenerative disorders [21, 23]. These aggregates are characteristic of neurodegenerative diseases, but are also features of disorders outside of the central nervous system, including protein aggregate myopathies (PAMs) [19]. One unifying hypothesis relating to the pathogenesis of these proteinopathies is the age-related disruption of the protein homeostasis system [21, 23]. For example, mutations in aggregation-prone proteins or changes in the cellular environment promote protein misfolding and subsequent aggregation in affected tissues [8, 12]. These aggregation events lead to further progressive impairment in protein surveillance and degradation pathways, causing further aggregation of other aggregation-prone proteins.

**Fig. 1:**
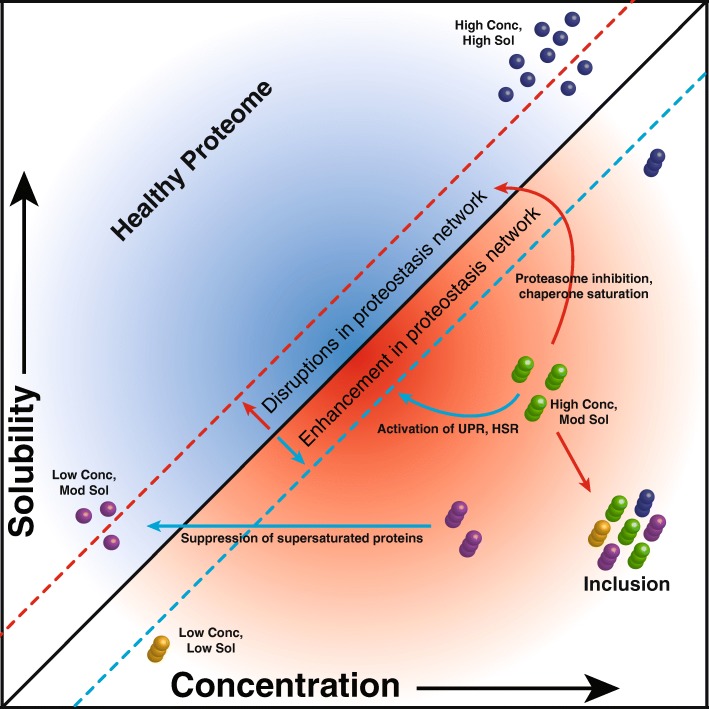
A protein aggregates when its concentration exceeds its solublity, thus becoming supersaturated. Supersaturated proteins tend to be abundantly expressed despite having a relatively low solubility. Disruptions in the protein homostasis system favor protein aggregate formation whereas an enhancement in protein homeostasis favors protein solubility (dashed lines). The translational repression of abundant aggregation-prone proteins favors a non-aggregated proteomic state. Protein inclusions in diseased tissue are heterogenous and composed of multiple proteins that have exceeded their solubility threshold

To rationalize these observations, we recently proposed that protein aggregation is a widespread phenomenon associated with the intrinsic supersaturation state of the proteome [3, 33]. Proteins become supersaturated when their cellular concentration exceeds their solubility, which is dictated by the physico-chemical characteristics of their amino acid sequences. Thus, supersaturation is a measure of the balance between concentration and solubility of proteins (Fig. 1). Upregulation of the heat shock response and the level of molecular chaperones or impairment in protein quality control can positively or negatively modulate the propensity of a protein to aggregate. This principle suggests that supersaturated proteins are most vulnerable to alterations in protein homeostasis [2, 9, 11, 12, 30, 31, 37, 41]. To measure protein supersaturation, we have developed a metric that combines a sequence-based prediction of aggregation propensity and estimates protein concentration from transcriptomic and proteomic data of thousands of human proteins [3]. With this approach, we reported that proteins found in inclusions in Alzheimer’s disease (AD), Parkinson’s disease (PD), and amyotrophic lateral sclerosis (ALS) have high supersaturation scores even in control tissues [3, 5, 22]. We have also similarly shown that the proteins that aggregate in aging *C. elegans* are supersaturated [3].

Remarkably, the enrichment for supersaturated proteins in neurodegenerative pathways is still detectable even when estimating supersaturation levels from average abundances across a wide variety of non-pathological tissues. However, the tissue selectivity of many protein conformational disorders suggests that the risk of misfolding may depend in part on the specific proteomic context. A limitation of previous studies on supersaturation is the absence of this context, because of the difficulty of obtaining living brain tissue from patients with neurodegenerative disease [8, 10]. Because muscle can be directly biopsied, the PAMs offer a means to determine how proteinopathies can remodel the proteome homeostasis in specific tissues, and whether changes in the metastable subproteome help to explain disease progression and pathology. In these degenerative muscle disorders, protein accumulates into inclusion bodies in affected myofibers [19, 40]. In some cases these inclusions contain the same proteins associated with neurodegenerative diseases, such as TDP-43 and SQSTM1 [40].

Most hereditary PAMs are due to dominantly inherited missense mutations in specific proteins resulting in their destabilization and subsequent aggregation [19]. By contrast, sporadic inclusion body myositis (IBM) is an acquired PAM with no clear genetic etiology manifesting exclusively in patients over 45 years of age [39]. Two types of pathological structures exist in PAMs: inclusion bodies, which are often immunoreactive for the mutated protein in the corresponding hereditary diseases, and rimmed vacuoles (RVs), which are pathological structures found in affected myofibers and containing aggregated proteins in association with degradative debris such as ubiquitin and autophago-lysosomal proteins [39]. In the present study, we use quantitative proteomic data from human patient tissues to test the hypothesis that supersaturation of a metastable subproteome explains protein inclusions in PAMs. Moreover, we explore how this metastable subproteome changes between healthy cells, diseased cells and inclusion-bearing cells.

## Results

### IBM-associated proteins are supersaturated in healthy tissues

**Fig. 2:**
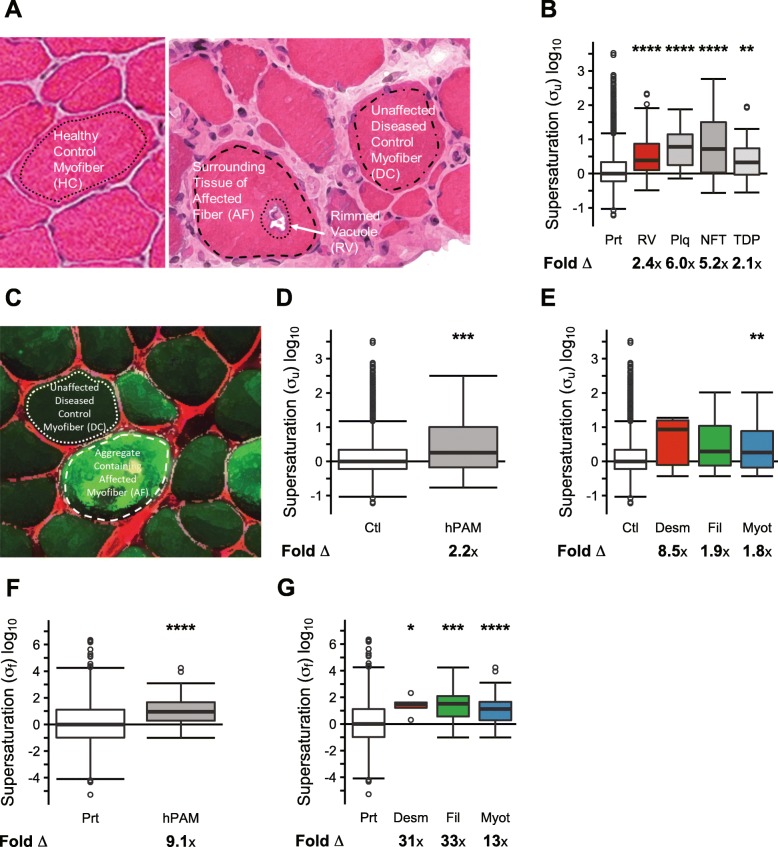
Proteins in rimmed vacuoles from protein aggregation myopathies are supersaturated. Representative images of: **(a)** healthy control myofibers (HC), control unaffected myofibers in diseased samples (DC), surrounding tissues of affected fibers (AF), and rimmed vacuoles (RV) from human subjects with inclusion body myositis, and **(c)** DC and AF samples from human subjects with myotilin mutations. Outlines represent areas for LMD. In **(c)**, prior to LMD, muscle was immunostained with an antibody directed to myotilin (green) to identify aggregate containing fibers (AF). **b**, **d**, **e** Comparison of the unfolded supersaturation scores (σ_u_) of the proteome (Prt) (*N* = 15,954) and (**b)** proteins enriched in RVs (RV) (*N* = 50), amyloid plaques (Plq) (*N* = 26), neurofibrillary tangles (NFT) (*N* = 76), proteins found in TDP-43 inclusions (TDP) (*N* = 32); **(d)** proteins enriched in affected fibers from any of three protein aggregation myopathies (hPAM) (N = 50); and **(e)** proteins enriched in affected fibers from individual protein aggregation myopathies involving desmin (Desm) (*N* = 6), filamin (Fil) (*N* = 16), and myotilin (Myot) (*N* = 46) mutations. **f**, **g** Comparison of the folded supersaturation scores (σ_f_) for the proteome (Prt) (*N* = 1605) and **(f)** the proteins enriched in affected fibers from any of three protein aggregation myopathies (hPAM) (N = 46) and **(g)** the proteins enriched in affected fibers from individual protein aggregation myopathies involving desmin (Desm) (N = 5), filamin (Fil) (N = 15), and myotilin (Myot) (*N* = 43) mutations. The fold change (Δ) represents the fold difference in the median σ_u_ or σ_f_ scores between each inclusion type and the proteome. The median σ_u_ or σ_f_ supersaturation score for the proteome is normalized to 0. Boxes range from the 25th percentile to 75th percentile, while whiskers extend to maximum and minimum data points up to 1.5x interquartile range above and below the limits of the boxes. Remaining outliers are plotted as open circles. Statistical significance determined by one-tailed Wilcoxon/Mann-Whitney test with Holm-Bonferroni correction. *p < 0.05, **p < 0.01, ***p < 0.001, ****p < 0.0001

We previously performed laser microdissection to collect areas of single fibers from muscle biopsies of 18 patients with IBM [14]. These samples were taken from normal healthy fibers, or in the case of IBM-affected muscles, from affected RV-containing fibers and adjacent normal appearing fibers. We then analyzed these samples by mass spectrometry using label-free spectral count-based relative protein quantification (see Methods). For the study presented here, healthy control and IBM proteomic datasets were generated from healthy control myofiber regions (HCs), unaffected myofiber regions from IBM patients (disease controls, DCs), non-vacuole containing sarcoplasmic regions of affected fibers (AFs), and myofiber regions containing rimmed vacuoles (RVs) (Fig. 2a).

Comparison of these datasets enabled us to identify a set of proteins enriched within RVs, as compared to DCs. This list of 53 RV-enriched proteins includes 17 proteins previously identified to accumulate in IBM tissue (Additional file 1: Dataset S1). We next asked whether these proteins share similar biophysical features despite their different sequences, structures and functions. We had previously estimated supersaturation of a protein as the product of its predicted aggregation propensity (given by the Zyggregator score (*Z*_*agg*_) which correlates negatively with its solubility) and its expression level, either based on mRNA levels from microarray data or proteomic analysis [3].

We thus compared the supersaturation levels of RV-enriched proteins to those of co-aggregators within amyloid plaques [24], neurofibrillary tangles [38], and TDP-43 inclusions [5] (Additional file 1: Dataset S2). As an approximation of the supersaturation level for a given protein, we used mRNA levels averaged over dozens of different human tissues unaffected by misfolding disease (Additional file 1: Dataset S4) and aggregation propensities predicted from the primary sequences for the unfolded states of proteins (*Z*_*agg*_) (Additional file 1: Dataset S3) termed the unfolded supersaturation score (σ_u_) (Additional file 1: Dataset S2). While this approach does not benefit from tissue specificity, it was previously shown that this average estimate demonstrated elevated supersaturation scores for proteins associated with aggregation and cellular pathways implicated in neurodegenerative disorders and enabled the direct comparison of inclusions from muscle to the central nervous system [3].

We found that proteins enriched in RVs have elevated supersaturation scores (σ_u_) in control tissues (RV, median fold change (Δ): 2.4x, *p* = 1.4•10^− 6^). This was similar to proteins observed to co-aggregate (co-aggregators) with plaques (median Δ: 6.0x, *p* = 4.5•10^− 8^) and neurofibrillary tangles (median Δ: 5.2x, *p* = 1.3•10^− 13^) in AD, and TDP-43 (median Δ: 2.1x, *p* = 1.8•10^− 3^) in ALS, respectively (Fig. 2b, Additional file 1: Dataset S7). The elevated supersaturation score for RV-enriched proteins was also present when we calculated tissue-specific supersaturation scores (${\upsigma}_u^{ts}$_)_ using the subset of the cross-tissue microarray expression database that included skeletal muscle expression (RV: median Δ: 2.1x, *p* = 2.2•10^− 6^) (Additional file 2: Figure S1; Additional file 1: Dataset S7). Comprehensive statistical results are shown in Additional file 1: Dataset S12.

### hPAM-associated proteins are supersaturated in healthy tissues

To determine whether the phenomenon of supersaturation observed for IBM-associated proteins (Fig. 2b), an acquired PAM, is also observed for proteins associated with hereditary PAMs (hPAM), we extended our studies to proteomic datasets from laser microdissected myofibers of muscle biopsies of patients with three different genetically defined hPAMs (10 patients with *DES* mutations, 7 patients with *FLNC* mutations and 17 patients with *MYOT* mutations) [20, 25, 26]. Samples were taken from affected aggregate-containing fibers (AF) or adjacent normal appearing disease control fibers (DC) (Fig. 2c). We then identified proteins that are enriched within the aggregate-containing fibers, as compared to unaffected disease control fibers (Additional file 1: Dataset S2). The σ_u_ score is similarly elevated for the proteins enriched in hPAM aggregate fibers (AF) (median Δ: 2.2, *p* = 6.9•10^− 4^) (Fig. 2d, Additional file 1: Dataset S7). We note, however, that sample size limitations led to statistically insignificant results for two of the three individual hPAMs (desminopathy median Δ: 8.5x, *p* = 9.8•10^− 2^; filaminopathy median Δ: 1.9x, *p* = 8.3•10^− 2^, myotilinopathy median Δ: 1.8x, *p* = 6.7•10^− 3^) (Fig. 2e). We then calculated ${\upsigma}_u^{ts}$ and found the increased supersaturation of proteins in aggregate-containing tissue is significant in this context (hPAM: median Δ: 4.5x, *p* = 1.2•10^− 8^; desminopathy median Δ: 11x, *p* = 2.5•10^− 2^; filaminopathy median Δ: 5.6x, *p* = 1.4•10^− 3^, myotillinopathy median Δ: 3.9x, *p* = 7.5•10^− 7^) (Additional file 2: Figure S1; Additional file 1: Dataset S7). In addition, we estimated the significance of the increase in supersaturation of the ${\upsigma}_u^{ts}$ scores relative to the σ_u_ scores (*p* < 1•10^− 6^).

Particular to our proteomic datasets, we can evaluate the degree of supersaturation within the context of the skeletal muscle proteome rather than abundances from mRNA levels in public databases. Thus, we asked whether aggregate-enriched proteins in hPAMs were supersaturated, based on their abundance in background of a healthy muscle proteome (Additional file 1: Dataset S6). To do this, we combined protein abundances derived from healthy control muscle using a version of the Zyggregator algorithm that weights residue-level aggregation propensities based on predictions of the relative burial of proteins after folding (${\mathrm{Z}}_{\mathrm{agg}}^{\mathrm{SC}}$), as described previously [32] (Additional file 1: Dataset S3). This estimate is termed folded supersaturation score (σ_f_) as compared with the previous estimate of the unfolded supersaturation score (σ_u_) (Additional file 1: Dataset S9). To directly compare these two estimates (σ_u_ and σ_f_), we calculated the σ_f_ score of proteins enriched in hPAM aggregate-containing fibers (median Δ 9.1x, *p* = 5.3•10^− 5^) (Fig. 2f compared with Fig. 2d). The elevated supersaturation score among proteins enriched in hPAM aggregate-containing fibers relative to HC is a result of both abundances and aggregation propensities higher than those of the proteome (Δ: 6.3x, *p* = 3.9•10^− 7^; ${\mathrm{Z}}_{\mathrm{agg}}^{\mathrm{SC}}$ Δ: 2.2x, *p* = 0.049) (Additional file 2: Figure S2A-B, Additional file 1: Datasets S3 and S9). Similarly, we found elevated σ_f_ scores when considering proteins enriched in aggregate-containing tissue specific to each hPAM (Fig. 2g, Additional file 1: Dataset S2) (desminopathy median Δ: 31x, *p* = 1.4•10^− 2^; filaminopathy median Δ: 33x, *p* = 8.8•10^− 4^; myotillinopathy median Δ: 13x, *p* = 5.7•10^− 5^).

**Fig. 3:**
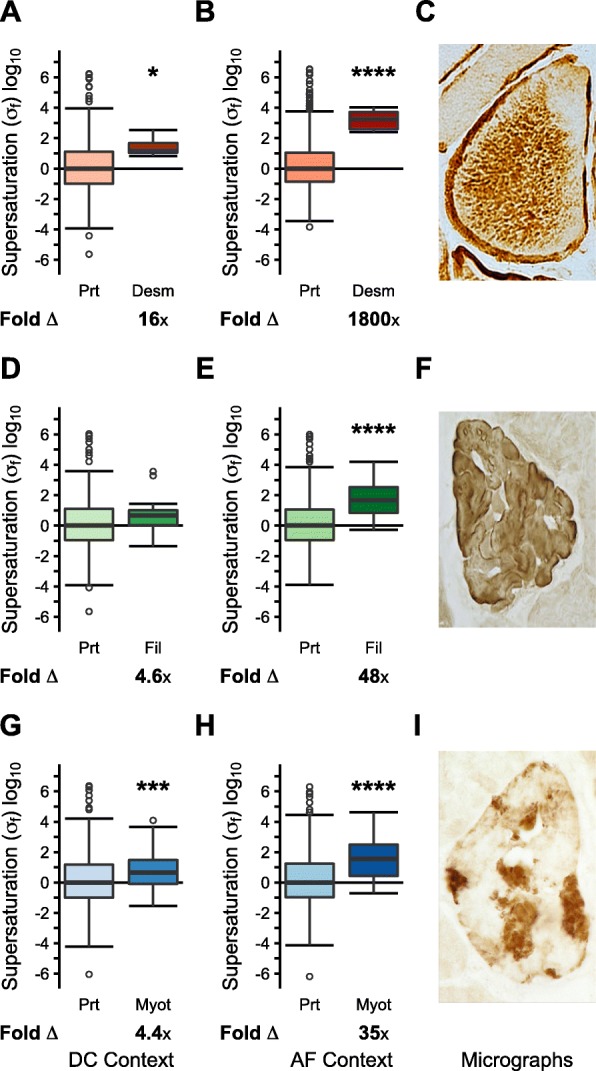
Protein supersaturation in hereditary protein aggregate myopathies. Comparison of folded supersaturation scores (σ_f_) between the proteome and proteins enriched in aggregate-containing myofibers (AF) for: **(a**, **b**, **c)** desminopathy (DC: Prt *N* = 611, Agg N = 6; AF: Prt *N* = 1387, Agg N = 6), **(d**, **e**, **f)** filaminopathy (DC: Prt *N* = 333, Agg N = 16; AF: Prt *N* = 507, Agg N = 16), and **(g**, **h**, **i)** myotillinopathy (DC: Prt *N* = 680, Agg N = 46; AF: Prt *N* = 742, Agg N = 46). Box plots and statistical tests as in Fig. 1. *p < 0.05, **p < 0.01, ***p < 0.001, ****p < 0.0001

In unaffected diseased control myofibers (DC), we found that the σ_f_ scores of proteins enriched in aggregate-containing myofibers (AF) were elevated relative to the proteome (desminopathy median Δ: 16x, *p* = 1.2•10^− 2^; filaminopathy median Δ: 4.6x, *p* = 5.7•10^− 2^; myotilinopathy median Δ: 4.4x, *p* = 2.7•10^− 3^) (Fig. 3a, d, g). In comparison, the σ_f_ scores of these proteins in affected fibers (AF) were higher (desminopathy median Δ: 1700x, *p* = 4.3•10^− 5^; filaminopathy median Δ: 48x, *p* = 4.2•10^− 5^; myotilinopathy median Δ: 33x, *p* = 3.6•10^− 8^) (Fig. 3b, e, h). To confirm the robustness of these results, we introduced varying amounts of random noise into our data, and found that the results are robust even when noise of at least 5 times the magnitude of the signal, and in many cases as much as 20x the magnitude of the signal, is introduced (Additional file 2: Figures S3-S4).

### Escalating supersaturation in IBM

**Fig. 4:**
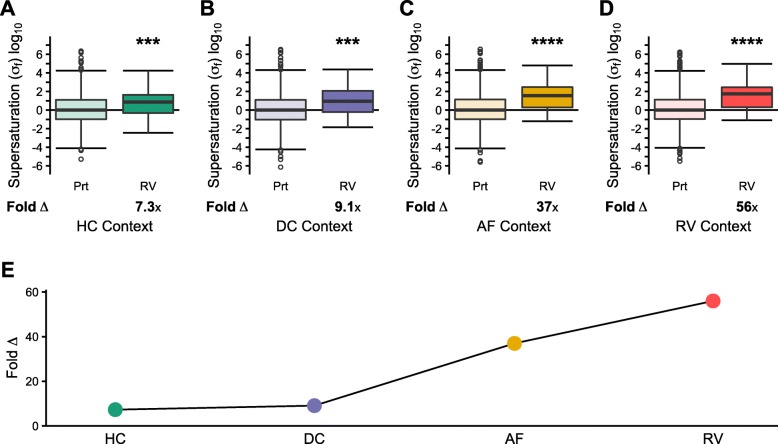
Escalating supersaturation in inclusion body myositis. Comparison of folded supersaturation scores (σ_f_) for the proteome (Prt) and proteins enriched in rimmed vacuoles (RV) relative to diseased control myofibers. **a** Healthy control myofiber (HC) (Prt N = 1605, RV *N* = 47), **b** control myofibers unaffected in diseased samples (DC) (Prt *N* = 1988, RV *N* = 52), **c** aggregate-containing affected myofibers (AF) (Prt *N* = 2396, RV N = 52), and **(d)** rimmed vacuoles (RV) (Prt *N* = 2104, RV N = 52). **e** Comparison of the fold difference in median σ_f_ between RV and Prt. Box plots and statistical tests as in Fig. 1. *p < 0.05, ***p < 0.001, ****p < 0.0001

We used our IBM proteomic datasets, to segment the data starting from healthy controls (HC) and continuing to unaffected fibers in affected patients (DC), areas from affected fibers surrounding the RV (AF), and the RV itself (RV) (Fig. 2a, Fig. 4a-e, Additional file 1: Datasets S6 and S9). By this approach, we were able to determine how the σ_f_ scores of the proteins that are enriched in RVs transition from healthy fibers to aggregate-containing fibers. We calculated σ_f_ based on protein abundances for each of these contexts. Even in healthy controls, the σ_f_ scores of RV-enriched proteins are higher in the muscle context than what we found in the cross-tissue transcriptional analysis for unfolded supersaturation (σ_u_) (median Δ: 7.3x, *p* = 9.6•10^− 4^) (comparison *p* < 2.0•10^− 5^) or with the skeletal muscle unfolded supersaturation score (${\upsigma}_{\mathrm{u}}^{\mathrm{ts}}$) (median Δ: 2.1x, *p* = 2.2•10^− 6^) (Fig. 4a compared with Fig. 2b and Additional file 2: Figure S1). In healthy control (HC) fibers, this result is driven by the higher median aggregation propensity of RV-enriched proteins rather than an increase in abundance (abundance Δ: 1.9x, *p* = 0.12; ${\mathrm{Z}}_{\mathrm{agg}}^{\mathrm{SC}}$ Δ: 7.4x, *p* = 3.7•10^− 3^) (Additional file 2: Figure S2C-D).

We found that σ_f_ increases with the physical proximity to the RVs (DC median Δ: 9.1x, *p* = 1.2•10^− 4^; AF median Δ: 37x, *p* = 1.2•10^− 9^; RV median Δ: 56x, *p* = 4.3•10^− 10^) (Fig. 4b-e). These results are robust against high levels of noise (Additional file 2: Figure S5-S6). In order to determine whether this rise in supersaturation was statistically significant, we performed a simulation of 1,000,000 trials in which we randomly selected 53 proteins to determine how frequently we could achieve the following pattern by chance: 1) elevated supersaturation for RV-associated proteins relative to the proteome in each proteome context (HC, DC, AF, and RV) and 2) a rising median Δ for the supersaturation of RV-associated proteins from HC to DC to AF to RV. In this way, we calculated a significance for achieving this pattern of escalating supersaturation *p* = 0.011 (see Methods). Given that this analysis included proteins expressed in some contexts but not in others (e.g. present in disease fibers but not healthy control fibers), we confirmed that the results were qualitatively similar when considering only a limited set of proteins expressed in all four contexts and had associated Zyggregator scores available (Additional file 2: Figure S7, Additional file 1: Dataset S9). To further validate these results, we performed these analyses utilizing aggregation predictions from the unfolded state with *Z*_*agg*_ and TANGO [7], which similarly demonstrated a significant escalation in supersaturation (Additional file 2: Figure S8-S9, Additional file 1: Dataset S10).

Like proteins enriched in RVs, proteins enriched in hPAM aggregate-containing fibers also exhibit an escalating σ_f_ in the sporadic disease context (Additional file 2: Figure S10). The escalation in σ_f_ is specific for proteins that accumulate in PAMs since proteins that co-aggregate with amyloid plaques (Additional file 2: Figure S11A-E) and neurofibrillary tangles (Additional file 2: Figure S11F-J) in AD do not exhibit escalating σ_f_ in IBM muscle tissues.

### RV proteins escape the downregulation of supersaturated proteins

**Fig. 5:**
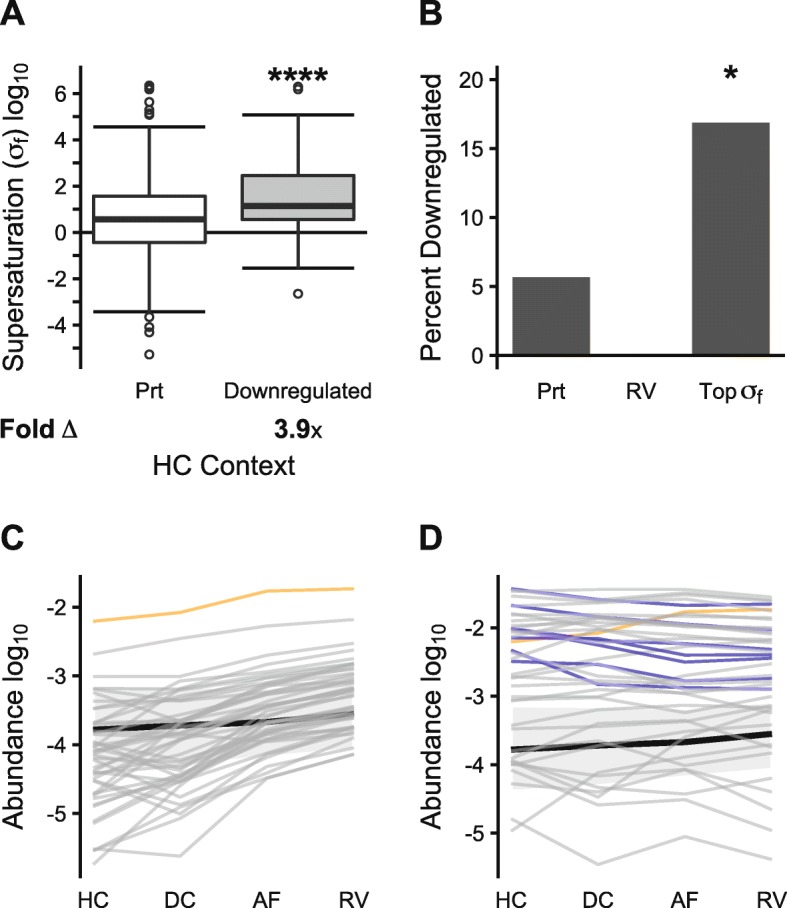
Protein supersaturation is associated with downregulation. In this analysis, only proteins that are detected in HC, DC, AF, and RV, and for which there are defined σ_f_ scores in HC are included. **a** Folded supersaturation scores (σ_f_) for the proteome (Prt) (*N* = 830) and proteins downregulated from HC to AF (N = 50). Box plots and statistical tests as in Fig. 1. **b** Percentage of proteins downregulated in the proteome (Prt) (48/830), proteins enriched in rimmed vacuoles (RV) (0/47), and top 5% most supersaturated proteins (based on HC context) (Top σ_f_) (7/41). Significance determined by the Fisher Exact test, with Holm-Bonferroni correction. **c** Protein abundances in HC, DC, AF, and RV are plotted for the 47 proteins enriched in RVs included in the subset analyzed in this figure. Desmin is highlighted in yellow, the only RV-enriched protein whose abundance is increased between HC and AF. **d** Protein abundances in HC, DC, AF, and RV are plotted for the 47 proteins with the highest supersaturation scores (top 5%). Desmin again is highlighted in yellow, the only highly supersaturated protein with rising abundance. Those proteins that are significantly downregulated between HC and AF are highlighted in blue. In **(c)** and **(d)**, the background black line and grey bar represent median and 25th–75th percentile range for the 830 proteins in the proteome in this subset. *p < 0.05, ****p < 0.0001

We recently reported a transcriptional suppression of supersaturated proteins and pathways in Alzheimer’s disease [4]. We therefore asked whether a similar phenomenon takes place at the transcriptional and translational levels in IBM. To do so, we determined the proteins differentially expressed in affected fibers (AF) relative to healthy controls in IBM (HC) (Additional file 1: Dataset S11). We found, across independent patient datasets, that 52 proteins are decreased and only one protein, desmin, is increased in affected fibers. Those proteins that are decreased in the surrounding fibers tend to have higher σ_f_ in healthy controls relative to the rest of the proteome (median Δ: 3.8x, *p* = 9.8•10^− 5^) (Fig. 5a). There are 830 proteins (Prt) in our dataset for which we had ${\mathrm{Z}}_{\mathrm{agg}}^{\mathrm{SC}}$ in HC context and abundance values across all four contexts, and of these, only 48 (5.8%) are decreased in abundance in affected fibers. By contrast, of the top 5% most supersaturated proteins in this subset (*N* = 41) (Top σ_f_), seven (17%) are decreased in affected fibers (enrichment *p* = 0.013) (Fig. 5b). As further validation of this phenomenon, we used RNA sequencing data from healthy muscle and IBM muscle to identify the transcripts of proteins that were downregulated in IBM tissue [15]. The downregulated transcripts correspond to proteins whose supersaturation scores tend to be elevated in healthy controls (median Δ: 2.6x, *p* = 3.3•10^− 3^) (Additional file 2: Figure S12A). There are 1366 transcripts for proteins in this dataset for which we were able to calculate σ_f_ in HC context. Of the top 5% most supersaturated proteins in this subset (*N* = 68) (Top σ_f_), 15 (22%) are decreased in expression in affected fibers versus 157 (11%) for the proteome (Prt) as a whole (enrichment *p* = 0.016) (Additional file 2: Figure S12B).

The RV-enriched proteins (RV) are an exception, as none of them is downregulated at the protein (Fig. 5b) or transcript (Additional file 2: Figure S12B) level. The individual abundance trajectories of these RV-enriched proteins trend towards rising abundances, although only one of the proteins, desmin, significantly rises in abundance between HC and AF (Fig. 5c). By contrast, among the top 5% most supersaturated proteins, there is a trend towards declining abundances, with a disproportionate number of proteins decreasing in abundance significantly in this group (Fig. 5d). These results suggest that supersaturated proteins are typically downregulated to control their abundance, but when they fail to be downregulated, they escape regulation and are more likely to deposit into inclusions.

As mentioned above, only one protein (desmin) is among the top 5% most supersaturated proteins, is an RV-enriched protein, and increases significantly in abundance. Mutations of desmin are associated with desminopathy, and this protein is found to be enriched in aggregate-associated tissues in myotilinopathy, filaminopathy and IBM. Thus, desmin represents the clearest example of an escape protein and is also highly associated with protein misfolding in muscle tissue.

## Discussion

By using protein abundance data from proteomic datasets derived from human biopsy specimens, we identified a metastable, supersaturated subproteome in muscle tissue from protein aggregate myopathies. These data are consistent with our previous studies that explored this phenomenon in proteinopathies associated with neuronal inclusions such as AD and ALS [3, 5, 9]. In contrast to those previous studies, the current analysis utilized the protein abundances from the affected tissues rather than estimated abundances averaged across tissues obtained from publicly available datasets. Thus, skeletal muscle offers a unique opportunity to explore how the proteome remodels during the course of aggregation-related disease, and the ways in which this can be rationalized by the physicochemical characteristics of solubility and expression. The proteins that are found in RVs and inclusions in disease have elevated supersaturation scores even in healthy tissue, suggesting that these proteins have an intrinsic risk for aggregation even in the normally expressed proteome.

Our ability to analyze samples taken from unaffected and affected myofibers within the same patient enabled us to demonstrate that the degree of supersaturation escalates from normal myofibers to unaffected diseased myofibers and finally to aggregate containing myofibers. In the case of IBM, the quality of the data made it possible to show an escalating supersaturation to the RV from surrounding tissue within the same fiber. To our knowledge, a confirmatory demonstration that a metastable subproteome increases in abundance from unaffected to affected cells in a vulnerable tissue from human biopsy specimens has never been performed before.

Our method enables an estimate of the supersaturation levels of thousands of proteins, based on expression levels and predicted aggregation propensities. Each of these underlying factors has certain limitations. While spectral counts have been shown to correlate with absolute protein abundance, this method is susceptible to biases related to the chemical properties of individual peptides and their likelihood of detection. The normalized spectral abundance factor corrects for one major aspect of such bias, protein length. The Zyggregator method has been evaluated most comprehensively on comparisons of point mutations in a given peptide [32]. Here, it is used to predict aggregation propensities of distinct proteins, for which it may be less accurate. In addition, we make aggregation propensity calculations based on canonical sequences, which neglectss the contribution of mutations or alternative splicing events in the propensity to aggregate of the eventual protein product. Finally, our method does not take into account the effects of subcellular localization, protein-protein interactions, or post-translational modifications. For these reasons, the approach is most useful in comparing groups of proteins, but may be limited in its accuracy for the supersaturation of any individual protein. Despite these limitations, we have demonstrated in a range of pathological contexts that supersaturation scores distinguish groups of aggregation-prone and conformational disease-associated proteins from the remainder of the proteome [3–5, 22].

**Fig. 6:**
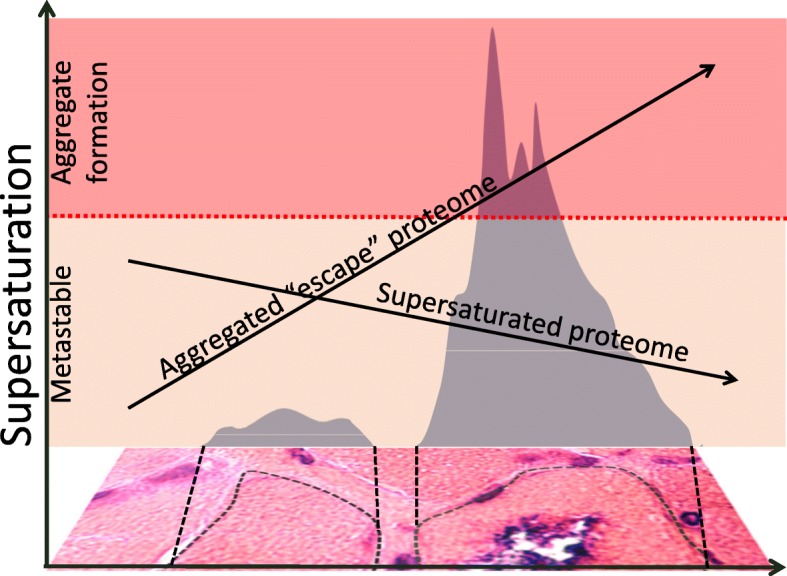
Escalating supersaturation of an aggregation-prone subproteome puts affected fibers at risk of inclusion formation in inclusion body myositis. 1) Supersaturation of the aggregate proteome increases to the point of aggregate formation at muscle inclusion bodies (gray regions). 2) The most highly supersaturated proteins decrease in abundance upon approaching the inclusion body. In contrast, the abundance of the aggregate/RV enriched supersaturated proteome increases and escapes downregulation

In both IBM and the three hereditary myopathies (hPAM) we studied, aggregate-associated proteins have elevated supersaturation scores in the context of healthy tissue (HC). In addition, affected fibers (AF) have higher relative supersaturation scores than unaffected fibers in patients known to have the disease (DC), both in the sporadic and hereditary cases. There are likely multiple factors contributing to the progressively rising abundance (and hence, supersaturation level) of proteins that deposit in RVs. In part, this may reflect a failure in proteostasis, as has been shown in a variety of protein conformational disorders [3]. Our results suggest that there may also be a failure to suppress the expression of some highly supersaturated proteins, given that those proteins that deposit in RVs run counter to a trend of declining abundance for supersaturated proteins (Fig. 6). The observation that this signal is already apparent at the transcriptional level favors a role for dysregulation of abundance, as opposed merely to a failure in the function of molecular chaperones or degradation machinery.

Our findings suggest that affected fibers have the capacity to downregulate their supersaturated proteome, and that this occurs at least in part at the transcriptional level. These data are consistent with our previous study in AD, in which downregulated proteins are similarly supersaturated relative to the proteome [4]. These results suggest that there may be a mechanism in IBM by which supersaturated proteins are preferentially downregulated to maintain their solubility. Indeed, the abundances of the top 5% most supersaturated proteins in skeletal muscle remained stable or decreased as they approached the RV. In contrast, supersaturated RV enriched proteins tended to increase in abundance.

These analyses identified one abundant protein, desmin, which was enriched within the RV and appears to escape the downregulation common to other highly abundant proteins. Desmin is a muscle specific type III intermediate filament (IF), and inherited missense mutations in this protein cause a PAM [13]. Desmin is abundantly expressed and requires multiple molecular chaperones to facilitate its proper assembly into the IF network that maintains the structural integrity of the sarcomere. In fact, dominant mutations in these molecular chaperones (e.g. CRYAB and DNAJB6) similarly lead to a PAM with prominent desmin aggregates [16, 36]. The formation of desmin IFs occurs via sequentially ordered steps that include dimer and tetramer formation, unit-length filament formation and filament elongation [1]. Some disease mutations affect IF assembly in vitro and in vivo resulting in cytosolic inclusions [1, 6]. We recently found that under physiologic conditions, desmin could also form amyloid fibrils [18]. The high abundance, reliance on molecular chaperones and ability to shift into an amyloidogenic state may explain why desmin escapes normal protein homeostasis regulation in PAMs. The behavior of desmin is exemplary of the close connection between the features that give rise to functional protein-protein interactions and pathological aggregation. We previously showed that the surfaces that mediate physiological protein-protein interactions tend to have a higher aggregation propensity than other protein surfaces [27]. Similarly, we have found that proteins that are involved in macromolecular complexes have higher supersaturation scores than the remainder of the proteome [3].

The pathogenic mechanism associated with supersaturation involves one or more of proteins reaching concentrations exceeding their solubility, thus resulting in aggregation [3]. Therapeutic approaches aimed at buffering this metastable proteome may be effective at reducing the degree of supersaturation. The present study identifies a common subset of abundant and aggregation-prone proteins from > 50 well characterized patients with PAMs. These proteins include amyloidogenic proteins such as gelsolin and TDP-43 that are not mutated in genetic PAMs but are mutated in hereditary amyloidosis and ALS, respectively. These findings suggest that therapies aimed at their reduction may also be effective at restoring protein homeostasis in PAMs. Our observation that desmin is both supersaturated in healthy control tissue and rises in abundance with the muscle’s pathological progression makes it a potential target for such intervention. Finally, the degree of supersaturation of a subset of proteins may serve as proxy for the proteostatic state of muscle. We envision using the concentration of the aggregate proteome as a biomarker in future therapies focused on PAMs. Taken together, our results indicate that the presence of supersaturated proteins represents a persistent challenge for the protein homeostasis system, and that failures in regulating the aggregation of these proteins leads to the formation of inclusions in a wide range of diseases, including neurodegenerative disorders and protein aggregate myopathies.

## Methods

### Datasets

The datasets used in this work and the proteins in each of them are described in Additional file 1: Datasets S1 and S2.

### Data analysis

Raw files were converted into the Mascot generic format (MGF) format using Proteome Discoverer 1.4 (Thermo Fischer Scientific, Germany). MGF files were searched against a combined database containing the Swiss-Prot part of the UniProt Knowledgebase (UniProtKB) [35] or *Homo sapiens* (release 2014/05/28, 20,265 curated entries). For the generation of shuffled decoy entries DecoyDatabaseBuilder was used [29]. Identifications were performed by Mascot 2.5 (Matrixscience Ltd., [28]) with a peptide mass tolerance of 10 ppm, fragment mass tolerance of 0.5 Da, one allowed missed cleavage and carbamidomethylation (C), oxidation (M) as well as phosphorylation (S, T, Y) as variable modifications. Label free relative quantification by spectral counting was performed as described in [14].

### Calculation of protein abundance

We previously reported abundances as spectral counts normalized by the total number of spectral counts in a given sample [14, 20, 25, 26]. Here, we performed an additional normalization step to account for the fact that longer proteins will generate more peptides in mass spectrometry than smaller proteins of the same abundance [17]. Akin to the normalized spectral abundance factor, we divided normalized spectral counts in our data sets by the protein length. We then divided these values by the sum of all such normalized values in a given sample. We then averaged these normalized protein abundances across replicates and log_10_-transformed these values to arrive at a final abundance value.

### Calculation of gene expression from microarray data

Microarray data was obtained from BioGPS pre-processed using gcrma as previously described. For cross-tissue analysis, cell ine and malignant tissue expression levels were excluded. Transcript identifiers were converted to UniProt IDs, with cases of ambiguous conversion or absence of reviewed UniProt IDs excluded from analysis. Values ≤0 were excluded. Expression levels were then log_10_-transformed then averaged across all values for a given UniProt ID. A similar procedure was done for the skeletal muscle analysis, but limiting it to the two arrays of skeletal muscle data.

### Calculation of gene expression from RNA sequencing data

Processed RNA sequencing data was obtained, with expression levels reported in FKPM (GEO Datasets GSE102138) [15]. Any values ≤0 were excluded. Identifiers were converted to reviewed UniProt IDs, with ambiguous conversions excluded from further analysis. In cases in which one multiple identifiers mapped to a single UniProt IDs, these FKPM values were averaged. The values were then log_10_-transformed. Significantly upregulated and downregulated transcripts were identified based on the reported q-values. In cases in which there were multiple q-values associated with a given UniProt ID, the largest q-value was used. The q-values reported were two-tailed, which we converted to one-tailed q-values for the purpose of our analysis. We used a threshold of significance of *p* < 0.05.

### Calculation of protein aggregation propensity

For the human proteome set, we calculated the *Z*_*agg*_*,* ${Z}_{agg}^{SC}$ and TANGO scores as previously described [7, 34]. For TANGO, we set the parameters at pH = 7.4, T = 310 K, and ionic strength = 0.1 M. The supersaturation score *σ* is calculated as the sum

1 $$ \sigma =C+Z $$

where C is the log_10_ of the concentration and *Z* is aggregation propensity score; the concentrations are derived from the protein abundance levels. In each dataset, values were recentered such that the median σ score for each database was 0.

### Identification of proteins enriched in disease-associated inclusions

In order to determine vacuole-enriched proteins in the IBM data set, we compared abundance values in the RV dataset to those in the DC dataset. For this analysis, we only included proteins that had a non-zero abundance in both the DC and RV datasets, which constituted a total of 1302 proteins. For these proteins, we performed a one-tailed paired t-test. We then used the Benjamini-Hochberg method to calculate q-values for each of these proteins, using as a threshold of significant q < 0.05, for a False Discovery Rate of 5%.

### Gaussian noise generation

We performed noise testing to evaluate the robustness of our results for the comparison of supersaturation scores among the IBM data sets, as well as the hPAM data sets. We defined one hundred noise levels on the basis of the standard deviation of a series of Gaussian distributions with mean of 0. The range of standard deviations was log_10_(1.1) to log_10_(10.1). At each noise level *l*, we performed 100 trials *t*, in which we drew a random number *n*_*l*, *t*, *p*_ from that the noise level distribution for each of the *p* proteins in the database. The noise-introduced supersaturation score *σ*_*p*, *l*, *t*_ was defined as

2 $$ {\sigma}_{p,l,t}={\sigma}_p+{n}_{l,t,p} $$

For trial *t* of noise level *l*, the set *S*_*l*, *t*_ of noise values is

3 $$ {S}_{l,t}=\left\{{n}_{l,t,1},{n}_{l,t,2},\dots, {n}_{l,t,p}\right\} $$

The set *m*_*l*, *t*_ of linear magnitudes of noise for trial *t* of noise level *l* is

4 $$ {m}_{l,t}=\left\{{10}^{\ln \left\lceil {n}_{l,t,1}\right\rceil },{10}^{\ln \left\lceil {n}_{l,t,2}\right\rceil },\dots, {10}^{\ln \left\lceil {n}_{l,t,p}\right\rceil}\right\} $$

For noise level *l*, the set *M*_*l*_ of median noise values for its constituent trials is

5 $$ {M}_l=\left\{ median\left({m}_{l,1}\right), median\left({m}_{l,2}\right),\dots, median\left({m}_{l,100}\right)\right\} $$

In each Gaussian noise plot, the values plotted on the x-axis were the median of *M*_*l*_ with error bars representing the standard error of the mean as calculated using default settings in the Python package SciPy.

### Gaussian noise significance testing

For each trial at each noise level, we determined the sets of noise-modified *σ* scores for the data sets under consideration. A one-tailed Wilcoxon/Mann-Whitney U test was performed for each of these trials, with multiple hypothesis correction performed based on the same families used for the original analysis, with one difference. At each noise level, the median of the *p*-values for the 100 trials was plotted with error bars representing the standard error of the mean as calculated using default settings in the Python package SciPy. We performed a one-sided one-sample t-test using the distribution of p-values for a given trial to test the null hypothesis that the mean of these p-values is not significantly less than 0.05. For those cases in which we could not reject the null hypothesis, we plotted the points in grey; otherwise, we plotted the points in color.

### Gaussian noise fold change testing

For each trial at each noise level, we determined the sets of noise-modified *σ* scores for the data sets under consideration. The linear difference *d*_*l*, *t*_ between the medians of the supersaturation scores of the control set *C*_*l*, *t*_ and experiment set *E*_*l*, *t*_ being tested at noise level *l* and trial *t* is

6 $$ {d}_{l,t}={10}^{median\left({E}_{l,t}\right)- median\left({C}_{l,t}\right)} $$

At noise level l, we plotted the median of set {*d*_*l*, 1_, *d*_*l*, 2_, …, *d*_*l*, 100_} with error bars representing the standard error of the mean as calculated using default settings in the Python package SciPy. We performed a one-sided one-sample t-test using the distribution of fold change values for a given trial to test the null hypothesis that the mean of these fold changes is not significantly greater than 1. For those cases in which we could not reject the null hypothesis, we plotted the points in grey; otherwise, we plotted the points in color.

### Overlap analysis

In Fig. 5b and Additional file 2: Figure S12B, the Fisher exact test is used to calculate enrichment of data sets for particular categories of proteins.

### Statistical significance of escalating supersaturation

To test the significance of our observations of rising supersaturation (Fig. 4, Additional file 2: Figures S7–11) we used a simulation. The null hypothesis was that it would arise by chance that 1) the median Δ > 0 for a set of proteins of interest in each context and 2) median Δ of those proteins would rise successively from HC to DC to AF to RV contexts. To test this, we performed the following procedure *K* times, where *K* =1,000,000. For each trial *k*, we randomly selected *N* proteins from the proteome (where *N* is equal to the number of proteins of interest, for instance 53 in the case of RV-enriched proteins or 51 in the case of hPAM-enriched proteins). When selecting *N*, we used the total number of proteins meeting a particular criterion, even if a smaller number of those proteins was actually present in the original dataset. For these *N* proteins, *D* is the set of median Δ compared to the proteome for each of the four contexts:

7 $$ D\equiv \left\{ med{\Delta }_{HC}, med{\Delta }_{DC}, med{\Delta }_{AF}, med{\Delta }_{RV}\right\} $$

If the supersaturation rose successively at each from HC to DC to AF to RV, and median Δ > 0 in each context, we assigned a score *E*_*k*_ of one; otherwise, we assigned a score *E*_*k*_ of zero. We then summed this score over the 1,000,000 trials.

8 $$ D=\left\{ med{\Delta }_{HC}, med{\Delta }_{DC}, med{\Delta }_{AF}, med{\Delta }_{RV}\right\} $$

9 $$ {E}_k=\left\{\begin{array}{c}1,\kern0.5em if\min (D)>0\  and\  med{\Delta }_{RV}> med{\Delta }_{AF}> med{\Delta }_{DC}> med{\Delta }_{HC}\\ {}0,\kern26em otherwise\end{array}\right.\kern0.5em $$

We estimated the significance of the escalation in supersaturation as follows:

10 $$ E=E1,\dots, EK $$

11 $$ p=\sum \limits_{k=1}^K\frac{E_k}{K} $$

12 $$ p=\sum \limits_{k=1}^K\frac{E_k}{K} $$

In order to test the isolated contribution of escalating median Δ, we removed the constraint of median Δ > 0, and calculated a score ${E}_k^r$:

13 $$ {E}_k^r=\left\{\begin{array}{c}1,\kern0.5em ifmed{\Delta }_{RV}> med{\Delta }_{AF}> med{\Delta }_{DC}> med{\Delta }_{HC}\\ {}0,\kern17.75em otherwise\end{array}\right. $$

14 $$ p=\sum \limits_{k=1}^K\frac{E_k}{K} $$

We considered all cases analyzed by our original constraints on family for the purpose of multiple hypothesis correction and all cases analyzed by the relaxed criteria a separate family. Multiple hypothesis correction was performed using the Holm-Bonferroni method. *P*-values for both constraints are reported in Additional file 1: Dataset S12.

### Statistical significance of comparative median Δ

To test the significance of differences in median Δ between different contexts (Figs. 2, 3 and 4), we used a simulation. The null hypothesis was that the difference in median Δ (*∆*_*∆*)_, of at least the magnitude reported would arise by chance. The reported difference in median Δ we refer to as ${\Delta }_{\Delta }^0$. To test this, we performed the following procedure *K* times, where *K* =1,000,000. For each trial *k*, we randomly selected *N* proteins from the proteome by the same procedure as above for escalating supersaturation. For these *N* proteins, we calculated the median Δ in contexts *C*_1_ and *C*_2_. Note that we performed this analysis in a one-tailed fashion.

15 $$ {S}_{\varDelta_{\varDelta }}=\left\{{\varDelta}_{\varDelta}^1,\dots, {\varDelta}_{\varDelta}^K\right\}{\varDelta}_{\varDelta }=\left\{{\varDelta}_{\varDelta}^1,\dots, {\varDelta}_{\varDelta}^K\right\} $$

where

16 $$ {\Delta }_{\Delta }^k= med{\Delta }_2^k- med{\Delta }_1^k $$

We assigned a score *E*_*k*_ to each trial and from all the trials together derived a *p*-value, as follows:

17 $$ {E}_k=\left\{\begin{array}{c}1,\kern0.5em {\Delta }_{\Delta }^k>{\Delta }_{\Delta }^0\\ {}0,\kern0.75em otherwise\end{array}\right. $$

18 $$ p=\sum \limits_{k=1}^K\frac{E_k}{K} $$

We considered all cases analyzed in this fashion as a single family. Multiple hypothesis correction was performed using the Holm-Bonferroni method. P-values are reported in Additional file 1: Dataset S12.

### Multiple hypothesis correction

In order to perform adequate multiple hypothesis correction while avoiding increasing Type II error by overcorrecting, it was necessary to group our results into a series of families on which multiple hypothesis correction would be performed meaningfully. We used the following principles to help divide the analyses in these studies into a set of coherent families. Except when they were being compared directly, hPAM and IBM data sets were considered part of separate families. IBM families were organized cross data subsets (that is, HC, DC, AF, and RV included in the same family). hPAM families were organized in three families: 1) HC, 2) DC, and 3) AF. This was organized in this way because there were multiple individual hPAMs, but analyses for the composite group of hPAM aggregate-enriched proteins could only be performed logically on the HC dataset as the other data sets were disease-specific. Analyses using σ_u_ were considered distinct from analyses using σ_f_. All σ_u_ analyses were considered as part of a single family. Among IBM data sets, we performed a series of analyses in which we compared σ_f_ levels between the proteome and particular subsets of proteins (RV-enriched, hPAM-enriched, plaque-enriched, NFT-enriched) across the four IBM data sets (HC, DC, AF, RV). We considered analyses involving each of these subsets as separate families. Additional file 1: Dataset S12 shows a summary of all statistical tests performed in this analysis, and groups those tests by their respective families.

### Laser microdissection (LMD) and sample processing

Patients provided informed consent. Study protocols were approved by the local ethics committee (reg. Number 3882–10) at Ruhr-University Bochum, Bochum, Germany. For each patient 250,000 μm^2^ of HC, DC, AF or RV tissue was collected by LMD (LMD 6500, Leica Microsystems, Wetzlar, Germany). Sample lysis and digestion were carried out as previously described [25]. Briefly, samples were lysed with formic acid (98–100%) for 30 min at room temperature (RT), followed by a sonication step for 5 min (RK31, BANDELIN electronic, Berlin, Germany). Samples were kept frozen at − 80 °C until digestion.

Prior to digestion the formic acid was removed and the collected samples were digested in 50 mM ammonium bicarbonate at pH 7.8. Samples were reduced and alkylated by adding dithiothreitol and iodoacetamide. Trypsin (Serva) was added to a final concentration of 1 μg. Digestion was carried out overnight at 37 °C and stopped by adding TFA to acidify the sample. Samples were purified using OMIX C18 Tips (Varian, Agilent Technologies, Böblingen, Germany) completely dried vacuum and again solved in 63 μl 0.1% TFA, as described in [25].

### Mass spectrometry

Sixteen microliter per sample were analysed by nano-liquid chromatography tandem mass spectrometry (nanoLC-ESI-MS/MS). The nano high performance liquid chromatography (HPLC) analysis was performed on an UltiMate 3000 RSLC nano LC system (Dionex, Idstein, Germany) as described in [26]. Peptides were separated with a flow rate of 400 nl/min using a solvent gradient from 4 to 40% B-solvent for 95 min. Washing of the column was performed for 5 min with 95% B-solvent and was then returned to 4% B-solvent. The HPLC system was online-coupled to the nano electrospray ionization (ESI) source of an Orbitrap elite mass spectrometer (Thermo Fisher Scientific, Germany). Mass spectrometric measurements were performed as previously described [14].

## Supplementary information

**Additional file 1: Dataset S1.** Proteins enriched in rimmed vacuoles. **Dataset S2.** Proteins enriched in plaques, neurofibrillary tangles, and protein aggregation myopathies. **Dataset S3.** Aggregation propensity scores. *Z*_*agg*_, ${Z}_{agg}^{SC}$, and TANGO scores (4) calculated as described in Methods. **Dataset S4.** mRNA expression levels. **Dataset S5.** Hereditary protein aggregation myopathy abundance data. **Dataset S6.** Sporadic inclusion body myositis abundance data. **Dataset S7.** Unfolded supersaturation scores. **Dataset S8.** Hereditary protein aggregation myopathy supersaturation scores (*σ*_*f*_). **Dataset S9.** Sporadic inclusion body myositis supersaturation scores (*σ*_*f*_). **Dataset S10.** Sporadic inclusion body myositis supersaturation scores (${\sigma}_f^T$). **Dataset S11.** Upregulated and downregulated proteins in sporadic inclusion body myositis. **Dataset S12.** Summary of statistical analysis and families of statistical tests.

**Additional file 2: Figure S1.** Unfolded skeletal muscle specific supersaturation for aggregated proteins from proteins enriched in rimmed vacuoles and hereditary protein aggregate myopathies. **Figure S2.** Both abundance and aggregation propensity contribute to the elevated supersaturation of aggregation-prone proteins. **Figure S3.** Fold changes for supersaturation estimates for aggregation-prone proteins in individual hereditary protein myopathies are robust against random noise. **Figure S4.** *P*-values for supersaturation estimates for aggregating proteins in individual hereditary protein myopathies are robust against random noise. **Figure S5.** Fold change for supersaturation estimates for IBM RV-enriched proteins are robust against random noise. **Figure S6.** *P*-values for supersaturation estimates for IBM RV-enriched proteins are robust against random noise. **Figure S7.** Escalating supersaturation in inclusion body myositis for proteins with coverage across sample types. **Figure S8.** Escalating supersaturation in inclusion body myositis using *Z*_*agg*_. **Figure S9.** Escalating supersaturation in inclusion body myositis using TANGO. **Figure S10.** Escalating supersaturation for hPAM aggregate-enriched proteins in the sporadic context. **Figure S11.** Plaque- and NFT-enriched proteins do not exhibit escalating supersaturation scores in IBM tissues. **Figure S12.** Protein supersaturation is associated with downregulation utilizing RNAseq datasets.
