# Supplementary material for: A metastable subproteome underlies inclusion formation in muscle proteinopathies
Source: Acta Neuropathol Commun. 2019 Dec 3;7:197. doi: 10.1186/s40478-019-0853-9 (PMC6891963; doi:10.1186/s40478-019-0853-9)
Supplement: Supplementary file 2 — Additional file 2: Figure S1. Unfolded skeletal muscle specific supersaturation for aggregated proteins from proteins enriched in rimmed vacuoles and hereditary protein aggregate myopathies. Figure S2. Both abundance and aggregation propensity contribute to the elevated supersaturation of aggregation-prone proteins. Figure S3. Fold changes for supersaturation estimates for aggregation-prone proteins in individual hereditary protein myopathies are robust against random noise. Figure S4. P-values for supersaturation estimates for aggregating proteins in individual hereditary protein myopathies are robust against random noise. Figure S5. Fold change for supersaturation estimates for IBM RV-enriched proteins are robust against random noise. Figure S6. P-values for supersaturation estimates for IBM RV-enriched proteins are robust against random noise. Figure S7. Escalating supersaturation in inclusion body myositis for proteins with coverage across sample types. Figure S8. Escalating supersaturation in inclusion body myositis using Zagg. Figure S9. Escalating supersaturation in inclusion body myositis using TANGO. Figure S10. Escalating supersaturation for hPAM aggregate-enriched proteins in the sporadic context. Figure S11. Plaque- and NFT-enriched proteins do not exhibit escalating supersaturation scores in IBM tissues. Figure S12. Protein supersaturation is associated with downregulation utilizing RNAseq datasets. [file 40478_2019_853_MOESM2_ESM.docx]

**Figure S1. Unfolded skeletal muscle specific supersaturation for aggregated proteins from proteins enriched in rimmed vacuoles and hereditary protein aggregate myopathies.** Comparison of skeletal muscle specific supersaturation scores ($\sigma_{u}^{ts}$) between the proteome and proteins enriched in rimmed vacuoles (RV) calculated using expression levels from microarray data obtained from skeletal muscle (Prt N=15944, RV N=50, hPAM N=49, desminopathy N=6, filaminopathy N=16, myotillinopathy N=45). Box plots and statistical tests as in **Figure 1**. *p < 0.05, **p < 0.01, ****p < 0.0001.

**Figure S2. Both abundance and aggregation propensity contribute to the elevated supersaturation of aggregation-prone proteins.** Comparison of the HC proteome (Prt) (N=1605) to (**a, b**) proteins enriched in affected fibers from any of three protein aggregation myopathies (hPAM) (N=46), or (**c**, **d**) proteins enriched in rimmed vacuoles (RV) (N=47). Results are provided in terms of: (**a**, **c**) protein abundance values estimated by mass spectrometry from healthy control myofibers, or (**b**, **d**) aggregation propensity scores (structurally corrected Zyggregator scores). Box plots and statistical tests as in **Figure 1**. *p < 0.05, **p < 0.01, ****p < 0.0001.

**Figure S3. Fold changes for supersaturation estimates for aggregation-prone proteins in individual hereditary protein myopathies are robust against random noise.** Random noise from increasingly wide Gaussian distributions was introduced into the protein supersaturation scores for the proteome and proteins enriched in affected fibers from hereditary myopathies, as shown in **Figure 2**. Points plotted are the mean ± S.E.M. of median fold difference between aggregate and proteome from 100 trials at each noise level based on: healthy control (**a-c**), disease control (**d-f**), or affected fiber context (**g-i**) for desminopathy (**a**, **d**, **g**), filaminopathy (**b**, **e**, **h**), and myotillinopathy (**c**, **f**, **i**), respectively. A one-tailed one-sample Student’s t-test was performed at each noise level to determine whether median fold differences were significantly greater than 1 **(d-f)**. Colored points represent significant results. Dashed line marks median fold difference of 1.

**Figure S4. P-values for supersaturation estimates for aggregating proteins in individual hereditary protein myopathies are robust against random noise.** Random noise from increasingly wide Gaussian distributions was introduced into protein supersaturation scores for the proteome and proteins enriched in affected fibers from hereditary myopathies, as shown in **Figure 2**. Points plotted are the mean ± S.E.M. of one-tailed Wilcoxon/Mann-Whitney p-values between aggregate and proteome from 100 trials at each noise level based on: healthy control (**a-c**), disease control (**d-f**), or affected fiber context (**g-i**) for desminopathy (**a**, **d**, **g**), filaminopathy (**b**, **e**, **h**), and myotilinopathy (**c**, **f**, **i**), respectively. A one-tailed one-sample Student’s t-test was performed at each noise level to determine whether p-values were significantly less than 0.05 **(d-f)**. Colored points represent significant results and grey points represent non-significant results. Dashed line marks p=0.05.

**Figure S5. Fold change for supersaturation estimates for IBM RV-enriched proteins are robust against random noise.** Random noise from increasingly wide Gaussian distributions was introduced into proteome and RV-enriched protein supersaturation scores shown in **Figure 3**. Points plotted are the mean ± S.E.M. of median fold difference between aggregate and proteome from 100 trials at each noise level based on: **(a)** healthy control, (**b**) disease control, (**c**) affected fiber, or (**d**) rimmed vacuole context. A one-tailed one-sample Student’s t-test was performed at each noise level to determine whether median fold differences were significantly greater than 1 **(d-f)**. Colored points represent significant results. Dashed line marks median fold difference of 1.

**Figure S6. P-values for supersaturation estimates for IBM RV-enriched proteins are robust against random noise.** Random noise from increasingly wide Gaussian distributions was introduced into proteome and aggregate protein supersaturation scores shown in **Figure 2**. Points plotted are the mean ± S.E.M. of one-tailed Wilcoxon/Mann-Whitney p-values between aggregate and proteome from 100 trials at each noise level based on: **(a)** healthy control, (**b**) disease control, (**c**) affected fiber, or (**d**) rimmed vacuole context. A one-tailed one-sample Student’s t-test was performed at each noise level to determine whether p-values were significantly less than 0.05 **(d-f)**. Colored points represent significant results and grey points represent non-significant results. Dashed line marks p=0.05.

**Figure S7. Escalating supersaturation in inclusion body myositis for proteins with coverage across sample types.** Comparison of supersaturation scores ($\sigma_{f}$ $\sigma_{f}$σ_f_) for the proteome (Prt N=830) and proteins enriched in rimmed vacuoles (RV N=47) relative to diseased control myofibers. In this analysis, only proteins detected in all IBM sample types (healthy control myofibers (HC), control myofibers unaffected in diseased samples (DC), aggregate-containing affected myofibers (AF), and rimmed vacuoles (RV)) are included. Supersaturation scores for: **(a)** HC, **(b)** DC, **(c)** AF, and **(d)** RV. **(e)** Comparison of the fold difference in median $\sigma_{f}$ $\sigma_{f}$σ_f_ between RV and Prt. Box plots and statistical tests as in **Figure 1**. **p < 0.01, ***p < 0.001.

**Figure S8. Escalating supersaturation in inclusion body myositis using**$Z_{\mathrm{agg}}$ ***Z_agg_*.** Comparison of supersaturation scores derived from protein abundances and$Z_{\mathrm{agg}}$ *Z_agg_* for the proteome (Prt) and proteins enriched in rimmed vacuoles (RV) relative to diseased control myofibers. **(a)** Healthy control myofiber (HC) (Prt N=1534, RV N=45), **(b)** control myofibers unaffected in diseased samples (DC) (Prt N=1883, RV N=50), **(c)** aggregate-containing affected myofibers (AF) (Prt N=2263, RV N=50), and **(d)** rimmed vacuoles (RV) (Prt N=2025, RV N=50). **(e)** Comparison of the fold difference in median $\sigma$ between RV and Prt. Box plots and statistical tests as in **Figure 1**. **p < 0.01, ****p < 0.0001.

**Figure S9. Escalating supersaturation in inclusion body myositis using TANGO.** Comparison of supersaturation scores ($\sigma_{f}^{T}$) derived from protein abundances and TANGO for the proteome (Prt) and proteins enriched in rimmed vacuoles (RV) relative to diseased control myofibers. **(a)** Healthy control myofiber (HC) (Prt N=1646, RV N=48), **(b)** control myofibers unaffected in diseased samples (DC) (Prt N=2050, RV N=53), **(c)** aggregate-containing affected myofibers (AF) (Prt N=2445, RV N=53), and **(d)** rimmed vacuoles (RV) (Prt N=2165, RV N=53). **(e)** Comparison of the fold difference in median $\sigma_{f}^{T}$ between RV and Prt. Box plots and statistical tests as in **Figure 1**. ***p < 0.001, ****p < 0.0001.

**Figure S10. Escalating supersaturation for hPAM aggregate-enriched proteins in the sporadic context.** Comparison of supersaturation scores ($\sigma_{f}$ $\sigma_{f}$σ_f_) calculated based on IBM context for the proteome (Prt) and proteins enriched in aggregates from hPAMs relative to diseased control myofibers (hPAM). Supersaturation scores and protein abundances for: **(a)** healthy control myofiber (HC) (Prt N=1605, hPAM N=46), **(b)** control myofibers unaffected in diseased samples (DC) (Prt N=1988, RV N=50), **(c)** aggregate-containing affected myofibers (AF) (Prt N=2396, RV N=50), and **(d)** rimmed vacuoles (RV) (Prt N=2104, RV N=50). **(e)** Comparison of the fold difference in median $\sigma_{f}$ $\sigma_{f}$σ_f_ between hPAM and Prt. Box plots and statistical tests as in **Figure 1**. ****p < 0.0001.

**Figure S11. Plaque- and NFT-enriched proteins do not exhibit escalating supersaturation scores in IBM tissues.** Comparison of supersaturation scores ($\sigma_{f}$ $\sigma_{f}$σ_f_) for the proteome (Prt) and proteins enriched in plaques (Plq, **a-d**) or neurofibrillary tangles (NFT, **f-i**). Supersaturation scores and protein abundances for: **(a, f)** healthy control myofiber (HC) (Prt N=1605, Plq N=16, NFT N=41), (**b, g)** control myofibers unaffected in diseased samples (DC) (Prt N=1988, Plq N=16, NFT N=42), **(c, h)** aggregate containing affected myofibers (AF) (Prt N=2396, Plq N=20, NFT N=48), and **(d, i)** rimmed vacuoles (RV) (Prt N=2104, Plq N=18, NFT N=48). Fold difference in median $\sigma_{f}$ between proteome and **(e)** Plq or **(j)** NFT. Box plots and statistical tests as in **Figure 1**. *p < 0.05, ****p < 0.0001.

**Figure S12. Protein supersaturation is associated with downregulation utilizing RNAseq datasets.** (**a,b**) Only proteins with defined $\sigma_{f}$ $\sigma_{f}$σ_f_ scores in HC for which transcripts were detected in both Ctl and sIBM RNA sequencing data are included. **(a)** Supersaturation scores ($\sigma_{f}$ $\sigma_{f}$σ_f_) for the proteome (Prt) (N=1366) and proteins downregulated from Ctl to sIBM RNA sequencing data (N=157). Box plots and statistical tests as in **Figure 1**. **(b)** Percentage of proteins downregulated in the proteome (Prt) (157/1366), amongst proteins enriched in rimmed vacuoles (RV) (0/42), and amongst the top 5% most supersaturated proteins (based on HC context) (Top σ_f_) (15/68).

**Dataset S1. Proteins enriched in rimmed vacuoles.** List of proteins enriched in rimmed vacuoles relative to unaffected fibers from diseased samples, with annotation as to whether they have previously been identified in IBM muscle, in what type of aggregate structure they have been found, and whether they have an associated mutation known to cause myopathy.

**Dataset S2. Proteins enriched in plaques, neurofibrillary tangles, and protein aggregation myopathies.** List of proteins enriched in plaques and neurofibrillary tangles or co-aggregating with TDP-43 are based on previously reported data (1-3). Proteins enriched in affected fibers from desminopathy, filaminopathy, and myotilinopathy also shown.

**Dataset S3. Aggregation propensity scores**. $Z_{agg}$, $Z_{agg}^{SC}$, and TANGO scores (4) calculated as described in Methods..

**Dataset S4. mRNA expression levels.** Cross-tissue mRNA, skeletal muscle mRNA microarray, control skeletal muscle RNA sequencing (FKPM), and sIBM RNA sequencing (FKPM) expression data.

**Dataset S5. Hereditary protein aggregation myopathy abundance data.** Protein abundance based on mass spectrometry data for desminopathy, filaminopathy, and myotilinopathy, collected and calculated as described in Methods.

**Dataset S6. Sporadic inclusion body myositis abundance data.** Protein abundance based on mass spectrometry data for sporadic inclusion body myositis, collected and calculated as described in Methods.

**Dataset S7. Unfolded supersaturation scores.** Supersaturation calculated using cross-tissue mRNA expression and $Z_{agg}$ (4) ($\sigma_{u}$ $\sigma_{f}$σ_u_), mRNA expression from skeletal muscle microarray and $Z_{agg}$ ($\sigma_{u}^{ts}$) and cross-tissue mRNA expression and TANGO ($\sigma_{u}^{T}$) as described in Methods.

**Dataset S8. Hereditary protein aggregation myopathy supersaturation scores (**$\boldsymbol{\sigma}_{\boldsymbol{f}}$**).** $\sigma_{f}$ based on mass spectrometry data and structurally-corrected aggregation propensity scores ($Z_{agg}^{SC}$) for desminopathy, filaminopathy, and myotillinopathy, calculated as described in Methods.

**Dataset S9. Sporadic inclusion body myositis supersaturation scores (**$\boldsymbol{\sigma}_{\boldsymbol{f}}$**).** $\sigma_{f}$ based on mass spectrometry data and structurally-corrected aggregation propensity scores ($Z_{agg}^{SC}$) for sporadic inclusion body myositis, calculated as described in Methods.

**Dataset S10. Sporadic inclusion body myositis supersaturation scores (**$\boldsymbol{\sigma}_{\boldsymbol{f}}^{\boldsymbol{T}}$**).** $\sigma_{f}^{T}$ based on mass spectrometry data and TANGO scores for sporadic inclusion body myositis, calculated as described in Methods.

**Dataset S11. Upregulated and downregulated proteins in sporadic inclusion body myositis.** Proteins upregulated and downregulated in sporadic inclusion body myositis affected fibers compared to healthy controls based on proteomic and RNA sequencing data.

**Dataset S12. Summary of statistical analysis and families of statistical tests.** Individual families of tests for the purposes of multiple hypothesis correction are shown under each bold heading, with corresponding figure to which the results are relevant. P-values are shown, with the method used to obtain the p-value, and the starred significance based on *p < 0.05, **p < 0.01, ***p < 0.001, ****p < 0.0001.

References

1. Ciryam P*, et al.* (2017) Spinal motor neuron protein supersaturation patterns are associated with inclusion body formation in ALS. *Proc Natl Acad Sci U S A* 114(20):E3935-E3943.

2. Liao L*, et al.* (2004) Proteomic characterization of postmortem amyloid plaques isolated by laser capture microdissection. *J Biol Chem* 279(35):37061-37068.

3. Wang Q*, et al.* (2005) Proteomic analysis of neurofibrillary tangles in Alzheimer disease identifies GAPDH as a detergent-insoluble paired helical filament tau binding protein. *FASEB J* 19(7):869-871.

4. Ciryam P, Tartaglia GG, Morimoto RI, Dobson CM, & Vendruscolo M (2013) Widespread aggregation and neurodegenerative diseases are associated with supersaturated proteins. *Cell Rep* 5(3):781-790.
